# Supplementary material for: A novel ALS-associated variant in UBQLN4 regulates motor axon morphogenesis
Source: eLife. 2017 May 2;6:e25453. doi: 10.7554/eLife.25453 (PMC5451210; doi:10.7554/eLife.25453)
Supplement: Supplementary file 1. — DOI: http://dx.doi.org/10.7554/eLife.25453.010 [file elife-25453-supp1.docx]

***UBQLN4* Sequencing Primers**

| UBQLN4_1F agtgttggaggcgcagaagccga | |
| --- | --- |
| UBQLN4_1R TTGCCTTCAGCTCCTCGGAGTCCCAG | |
| UBQLN4_2F cccatgactagagatgagagcaag | |
| UBQLN4_2R CTCTGGGTACAGTTAAGCAATGAG | |
| UBQLN4_3F gggttaaaatagttgtcccatagc | |
| UBQLN4_3R GTATCAATGTCTGGAACTCTGTCATGG | |
| UBQLN4_4F gaattcagtggctctggctaag |  |
| UBQLN4_4R CACGAATGAACAAATCAATGTAGG | |
| UBQLN4_5F cctggtcctgatcctgatacagac | |
| UBQLN4_5R TACTGTTGAGAACTGCCTTCAAGC | |
| UBQLN4_6F gctattaagtgagaggagagaatgg | |
| UBQLN4_6R CAAAATCCTGGAGCAGTATGAAC | |
| UBQLN4_7F tcattcggtgtctgaagtaaggtag | |
| UBQLN4_7R CTCCACAGTGTTTTGACCACAAG | |
| UBQLN4_8-9F gggaattcttagggtgaaaaggc | |
| UBQLN4_8-9R AGAGAGAAGAGTTGAAGGGGAGAAG | |
| UBQLN4_10F cacattctcatccctttcttgg |  |
| UBQLN4_10R CTATTCTATTGCTTCCCTTGAAACC | |
| UBQLN4_11F tgctggtcttagaaacccttaattc | |
| UBQLN4_11R AGAAGAACCGAATGCTGACATC | |
